# *Plasmodium vivax* liver stage assay platforms using Indian clinical isolates

**DOI:** 10.1186/s12936-020-03284-8

**Published:** 2020-06-22

**Authors:** Pradeep A. Subramani, Neha Vartak-Sharma, Seetha Sreekumar, Pallavi Mathur, Bhavana Nayer, Sushrut Dakhore, Sowmya K. Basavanna, Devaiah M. Kalappa, Ramya V. Krishnamurthy, Benudhar Mukhi, Priyasha Mishra, Noriko Yoshida, Susanta Kumar Ghosh, Radhakrishan Shandil, Shridhar Narayanan, Brice Campo, Kouichi Hasegawa, Anupkumar R. Anvikar, Neena Valecha, Varadharajan Sundaramurthy

**Affiliations:** 1 National Centre for Biological Sciences (NCBS), Tata Institute of Fundamental Research (TIFR), Bellary Road, Bangalore, 560065 India; 2 Institute for Stem Cell Biology and Regenerative Medicine (inStem), Bangalore, India; 3 Institute for Integrated Cell-Material Sciences (iCeMS), Institute for Advance Studies, Kyoto University, Yoshida-Ushinomiya-cho, Sakyo-ku, Kyoto, 606-8501 Japan; 4 ICMR-National Institute of Malaria Research (NIMR), Indian Council of Medical Research, Bangalore, India; 5 ICMR-National Institute of Malaria Research (NIMR), Indian Council of Medical Research, New Delhi, India; 6 Foundation for Neglected Disease Research, Bangalore, India; 7 Medicines for Malaria Venture, Geneva, Switzerland; 8 Manipal Academy of Higher Education, Manipal, 576104 Karnataka India

**Keywords:** Malaria, *Plasmodium vivax*, Induced pluripotent stem (iPS) cells, Malaria liver stage, Hypnozoite, Assay development, Indian isolates

## Abstract

**Background:**

Vivax malaria is associated with significant morbidity and economic loss, and constitutes the bulk of malaria cases in large parts of Asia and South America as well as recent case reports in Africa. The widespread prevalence of vivax is a challenge to global malaria elimination programmes. Vivax malaria control is particularly challenged by existence of dormant liver stage forms that are difficult to treat and are responsible for multiple relapses, growing drug resistance to the asexual blood stages and host-genetic factors that preclude use of specific drugs like primaquine capable of targeting *Plasmodium vivax* liver stages. Despite an obligatory liver-stage in the *Plasmodium* life cycle, both the difficulty in obtaining *P. vivax* sporozoites and the limited availability of robust host cell models permissive to *P. vivax* infection are responsible for the limited knowledge of hypnozoite formation biology and relapse mechanisms, as well as the limited capability to do drug screening. Although India accounts for about half of vivax malaria cases world-wide, very little is known about the vivax liver stage forms in the context of Indian clinical isolates.

**Methods:**

To address this, methods were established to obtain infective *P. vivax* sporozoites from an endemic region in India and multiple assay platforms set up to detect and characterize vivax liver stage forms. Different hepatoma cell lines, including the widely used HCO4 cells, primary human hepatocytes as well as hepatocytes obtained from iPSC’s generated from vivax patients and healthy donors were tested for infectivity with *P. vivax* sporozoites.

**Results:**

Both large and small forms of vivax liver stage are detected in these assays, although the infectivity obtained in these platforms are low.

**Conclusions:**

This study provides a proof of concept for detecting liver stage *P. vivax* and provide the first characterization of *P. vivax* liver stage forms from an endemic region in India.

## Background

The Global Technical Strategy for Malaria 2016–2030 has set major ambitious targets for malaria eradication, namely, the elimination of malaria from at least 35 countries and reduction of malaria incidence and mortality by at least 90% by 2030 [1]. Such global efforts are constantly challenged by relapsing malaria species and the risk of expanding drug resistance. Despite such challenges, malaria related deaths have showed a decline over the past decade, owing to the reduction in the number of *Plasmodium falciparum* malaria cases, which is mainly due to the fact that the national malaria control programmes in the past have always mainly focused on the most pathogenic and virulent form, *P. falciparum* [2] and the predominance of falciparum malaria within the African continent. Currently, *Plasmodium vivax* is responsible for 7.5 million malaria cases worldwide, causing equally debilitating disease as *P. falciparum*, and hence increasingly recognized as the biggest hurdle in malaria eradication [3]. Several factors contribute towards the additional challenges posed by *P. vivax*; (i) the difficulty to detect *P. vivax* infection due to its ability to circulate in the blood at very low levels, (ii) the ability to transmit prior to drug treatment, and most importantly, (iii) its ability to remain dormant inside the patient’s liver as hypnozoites, which depending on the strain, have the ability to reactivate several weeks, months, or years after the primary infection to produce relapsing forms of clinical disease [4]. An additional complication is the emergence of drug-resistant forms of the species, forcing certain malaria endemic countries to abandon chloroquine, the go-to drug for *P. vivax* treatment [5]. *Plasmodium vivax* might also have developed resistance to sulfadoxine-pyrimethamine (SP) and other anti-malarial drugs, such as mefloquine, due to point mutations in DHFR and DHPS genes concomitant to a substantial selective pressure exerted by SP treatment against *P. falciparum* [6]. The treatment strategies for vivax malaria are further complicated due to the need to use combinatorial drugs targeting both the blood stage and the dormant liver stage of the parasite. The only hypnozoicidal licensed drugs of 8-aminoquinoline class in the market, Primaquine, has many undesirable side-effects and is contraindicated in pregnant woman, children under 6 months of age and in patients with glucose-6-phosphate dehydrogenase (G6PD) deficiency, making it unsuitable for mass administration [7]. More recently, a new 8-aminoquinoline drug tafenoquine with a single dose radical cure regimen has been approved but the inherent problem remains [8, 9]. Therefore, there is an urgent need for development of new class of drugs acting on vivax liver stages [8].

A crucial target for drug development against *P. vivax* is the obligatory liver stage in the plasmodium life cycle, which ranges between 6 and 8 days and is characterized by a feature that distinguishes vivax from falciparum infection, the formation of hypnozoites that are responsible for multiple relapses post infection [10, 11]. The mechanisms of formation and re-activation of hypnozoites are poorly understood, largely owing to the difficulties associated with studying vivax biology. *Plasmodium vivax* has been refractory to several attempts to culture it in vitro [12, 13], thus restricting the studies to freshly obtained clinical isolates. Furthermore, the liver stage studies are hampered by the low level of infection typically seen in hepatocyte-like cells in culture in vitro [14–17], although recent advances in development of novel platforms have led to significant improvement in infectivity rates [18–21]. So far, hepatoma cell lines such as HC04 cells and primary human hepatocytes (PHH) have been used as in vitro models for screening and testing drugs targeting liver-stage malaria. PHH have the advantage of providing physiological context, better maintenance of metabolic activity and likely better predictive metrics for infection, but is highly variable depending on donor genetic background [18–20]. The hepatocyte-like cell lines such as HC-04 has distinct cost advantage and offers unlimited growth, but supports relatively low levels of infection [12, 14]. It is desirable to develop additional assay systems that could combine the advantages of these systems to support *P. vivax* liver stage growth. Hepatocytes differentiated from induced pluripotent stem cells (iPSCs) [22] could provide an alternative host cell source. Indeed, iPSC-derived hepatic cells have recently been used for modelling infectious liver diseases such as Hepatitis C virus and Hepatitis B virus [23, 24], as well as *P. vivax* [15, 25]. Compared to primary human hepatocytes that are sourced from a limited pool of individuals and have limited growth in vitro, iPSC-derived hepatocytes represent a more diverse genotype and an unlimited supply, and can be derived from blood or skin cells of any individual without the need for liver biopsy.

India is listed as a high malaria burden country [3] with vivax malaria being the most predominant form of malaria in India [2]. About half of all *P. vivax* cases globally are from India [3], yet very little is known about the liver stage vivax characteristics from Indian isolates. In this work, we report the establishment of a reproducible method for generation of *P. vivax* sporozoites from Indian clinical isolates, infection conditions in multiple platforms, including different hepatoma cell lines, primary human hepatocytes and *vivax* patient derived iPSC generated hepatocytes. To achieve this, the following processes were put in place; (i) production of infective *P. vivax* sporozoites from mosquitoes fed on blood of *P. vivax* patients in the vivax endemic region of Mangalore, Karnataka, and (ii) evaluation of infectivity using multiple assay platforms. This study provides a proof of concept for establishment of platforms for detecting *P. vivax* liver stage forms and provide the first characterization of *P. vivax* liver stage forms from a vivax endemic region in India.

## Methods

### Ethics statement

All experiments using patient cells were carried out as approved by Institutional Ethics Committee of ICMR-National Institute for Malaria Research (NIMR) and Health Ministry Screening Committee of Indian Council of Medical Research (ICMR). All stem cell experiments were carried out under Institutional Committee of Stem Cell Research, Institute for Stem Cell Biology and Regenerative medicine (inStem), and the National Apex Committee for Stem Cell Research (NAC-SCR). All experiments of transfection and infection were carried out after approval of Institutional Biosafety and Bio-Ethics Committee of NCBS and InStem.

### *Plasmodium vivax* patient screening and blood collection

Patient screening was conducted at Wenlock District Government Hospital, Mangalore by NIMR. A written informed consent was obtained from each patient participating in this study. *Plasmodium vivax* mono-infected patients were screened from patients diagnosed with malaria by Giemsa-staining of blood smears and Falcivax^®^ rapid diagnostic test with *P. vivax* specific lactate dehydrogenase. Additionally, the patients were confirmed to be negative for HIV and HCV. For mosquito blood feeding, the *P. vivax* infected blood samples were directly transferred into standard membrane feeding cup maintained at 37 °C.

### Production and isolation of *Plasmodium vivax* sporozoites

*Anopheles stephensi* mosquitoes were reared as described previously [26]. Briefly, the mosquitoes were maintained at 27 °C and 75–80% humidity with a 12 h light–dark cycle. Larvae were reared on yeast and dog biscuit in water (70:30 Brewer’s yeast : dog food (Pedigree brand; chicken and vegetable mixed). Pupae were segregated for adult emergence, and freshly emerged adult mosquitoes were fed on 10% d-glucose solution (Sigma-Aldrich) containing 0.05% para-aminobenzoic acid (PABA)(Sigma Aldrich) [27] and 10 µg/ml penicillin–streptomycin antibiotic cocktail (Invitrogen). For egg production, adult female were allowed to take non infected human blood through membrane feeding. To infect the aseptic mosquitoes with *P. vivax*, freshly collected *P. vivax* mono-infected venous blood in anticoagulant heparin sulfate-coated vacutainers (BD Bioscience) were either directly fed or washed the packed *P. vivax* infected red blood cells with AB + ve human serum (haematocrit adjusted to 50%), prior to feeding 3–4 days old female mosquitoes using membrane feeding apparatus maintained at 37 °C using a circulating water bath. Subsequently, the fully engorged mosquitos were transferred to arthropod containment facility and maintained at temperate conditions as described above. Subsequently, the mosquitoes were fed with water containing 10 µg/mL penicillin–streptomycin in 10% d-glucose to prevent the risk of contamination in the later hepatocyte infection assays [28, 29]. The infection and development of *P. vivax* in the mosquitoes was confirmed by detection of oocysts in the mid gut of the mosquitoes using 1% mercurochrome staining at day 8–10 post infection as described previously [30]. 14 days post blood feeding, salivary glands of the infected mosquitoes were dissected and mature *P. vivax* sporozoites were released by mechanical rupture. Insectary operations were carried out in NIMR, Bangalore.

### Preparation of primary human hepatocyte (PHH) monolayers

Cryopreserved Primary Human Hepatocyte (PHH) from Bio IVT, formerly Bioreclamation IVT, USA was used in all the studies [18]. Characterized PHH cells (UG4) that are known to be infected by *P. vivax* [18] were used. The cells were thawed according to manufacturer’s instructions and cell viability was assessed by trypan blue exclusion. The PHH cells were diluted using in vitro growth CP media (Bio IVT) containing the following antibiotics: Penicillin-50U/ml, Streptomycin-50 µg/ml and Neomycin-100 µg/ml. Cells were seeded onto Pre-collagen coated 384 well plates [18] at a density of 20,000 cells/well. In some experiments, hepatocytes were incubated with additional broad-spectrum antibiotic Moxifloxacin at 0.5-2 µg/ml and Amphotericin B at 2 µg/ml concentration for various periods to prevent early contamination and increase longevity of plated hepatocytes. The plates are incubated at 37 °C, 5% CO_2_ to develop confluent PHH cell monolayers prior to infection.

### HC04 cell line seeding and culture

HC04 (14,16) and other hepatoma-like cell lines tested (HepG2, Chang Liver, WRL-68, PLC/PRF) were maintained in MEM/F12 medium with 1 × anti-anti (Thermo Fisher) at 37 °C, 5% CO_2_. Viable cells quantified by trypan blue exclusion. HCO4 cells were seeded at a density of 25,000 cells per well in 384 well plate while other cells mentioned were seeded at 20,000 cells per well in 384 well plate or 80,000 cells per well in 96 well plate before infection.

### iPSC derivation from vivax patient PBMC

Three vivax patients’ PBMCs were processed for iPSC generation using CytoTune^®^-iPS 2.0 Sendai Reprogramming Kit (Thermo Fisher Scientific, Waltham, MA, USA) according to manufacturer’s Instruction. Briefly, the PBMCs were thawed, cultured for 5 days in a selection media to enrich CD34+ hematopoietic progenitor cells, and transfected with reprogramming factors, namely *Oct4*, *Sox2*, *Klf4* and *cMyc* as well as the reporter of transfection (GFP) gene in Sendai virus non-genome integrated vectors, to generate iPSCs. After 16 days of culture in iPSC culture medium, iPSC colonies emerged. The colonies were then cloned and expanded to establish the iPSC lines. At least three morphologically relevant and stable iPSC lines per patient were established from each PBMCs. Non-patient iPSC lines were derived from commercially available PBMCs from a healthy donor (AccuCell, Frederick, MD, USA). The iPSCs were expanded and stocked after confirmation of pluripotency marker expression and normal karyotype.

Nine patient iPSCs (BG1, BG2 and BG3 iPSC line from patient 1; NG1, NG3 and NG4 iPSC lines from patient 2; and S2, S4 and S5 iPSC lines from patient 3), non-patient iPSCs (K iPSC line from commercially available PBMCs) and H9 ESC (WA09, WiCell, Madison, WI, USA) were routinely cultured using standard feeder-dependent or feeder-free conditions as described previously [31]. Briefly, in feeder-dependent condition, cells were cultured in ESC media (DMEM/F12 medium) supplemented with 20% Knockout Serum Replacement (Thermo Fisher Scientific), MEM nonessential amino acids (Thermo Fisher Scientific), l-glutamine (Thermo Fisher Scientific), penicillin/streptomycin (Thermo Fisher Scientific) and bFGF (4 ng/ml; Thermo Fisher Scientific) on mitotically inactivated mouse embryonic fibroblasts (MEFs). In feeder-free condition, the cells were cultured on hESC-verified Matrigel (Becton, Dickinson and Company) coated plates in mTeSR1 media (Stem Cell Technologies, Vancouver, Canada). The feeder free condition was used for at least two to three passages prior to hepatic differentiation. Absence of mycoplasma contamination in all cells was confirmed at the time of freezing, after thawing and after every 2 months of culture by MycoAlert Mycoplasma Detection Kit (Lonza, Basel, Switzerland).

### Characterization of iPSCs

Pluripotency marker expression and normal karyotype was confirmed as described previously [31]. Briefly, the cells were fixed with 4% paraformaldehyde (PFA), washed with PBS, permeabilized with 0.1% Triton-X 100/PBS and blocked with 2% BSA/PBS. The cells were then incubated with primary antibodies: NANOG (4903S, Cell Signaling, Danver, MA,USA, 1:100), SOX2 (Y17, sc- 17320, Santa Cruz, Dallas, TX, USA, 1:100), OCT4 (C10, sc-5279, Santa Cruz, 1:100), SSEA3 (631, sc-21703, Santa Cruz, 1:100), SSEA4 (MC813, sc-59368, Santa Cruz, 1:100), TRA-1-60 (sc-21705, Santa Cruz), TRA-1-81 (sc-21706, Santa Cruz), followed by incubation with Alexa Fluor 594- or Alexa Fluor 488- conjugated secondary antibody (Thermo Fisher Scientific), and mounted with Vectashield with DAPI (Vector Laboratory, Burlingame, CA, USA). Alkaline phosphatase activity was detected by Vector Blue Alkaline Phosphatase Substrate Kit (Vector Laboratory) according to the manufacturer’s protocol. For karyotype analysis, the cells were arrested in metaphase with colcemide (KaryoMAX, Sigma-Aldrich, St. Louis, MO, USA), dissociated with trypsin/EDTA, treated with a hypotonic solution, and fixed with Carnoy’s fixative solution. Chromosomal G-bands were stained with Leishman’s staining solution (L6254, Sigma Aldrich).

### Hepatocyte differentiation from iPSCs and characterization

iPSC’s were differentiated to hepatocytes by step-by-step sequential differentiation protocol with oxygen control. Directed differentiation was achieved by sequential exposure to Activin A (120-14E, Peprotech, Rehovot, Israel) at 20% O_2_ to differentiate to definitive endoderm (DE stage), BMP4 (bone morphogenic protein 4, PHC 9534, Invitrogen), FGF2 (100-18B, Peprotech) HGF (100-39, Peprotech) at 4% O_2_ for hepatoblast (HB) and hepatocyte (HC) differentiation, and finally with Dexamethasone (D-2925, Sigma) and OncostatinM (OSM, 295-OM, R & D Systems, Minneapolis, MN, USA) at 20% O_2_ for hepatocyte maturation. Maturation of hepatocytes was further achieved via treatment with free fatty acids, high density lipoprotein and small molecules FH1 and FPH133, under ambient oxygen. At each stage mentioned, differentiation was confirmed by morphology, immunostaining and RT-qPCR with stage specific markers as described previously [31]. All specific probe/primes for RT-qPCR are TaqMan gene expression and assays performed following the manufacturer’s instructions (Applied Biosystems, Foster City CA, USA); OCT4 (POU5F1) (Hs01895061_u1), NANOG (Hs02387400_g1), SOX17 (Hs00751752_s1), GATA4 (Hs00171403_m1), FOXA2 (Hs00232764_m1), HHEX (Hs00242160_m1), HNF4A (Hs00230853_m1), PROX1 (Hs00896294_m1), AFP (Hs00173490_m1), ALB (Hs00910225_m1) and A1AT (SERPINA)(Hs01097800_m1). The primary antibodies used for Immunostaining were; FOXA2 (AB4125, Millipore, Billerica MA, USA), SOX17 (AF1924, R&D Systems), CXCR4 (MAB173, R&D Systems), HNF1b (C-20, Santa Cruz), HNF4a (H1415, R&D Systems), AFP (ab75705, Abcam, Cambridge, UK), A1AT (ab17438, Abcam, Cambridge, UK), ALB (MAB1455, R&D Systems), CK18 (SAB3300015, Sigma-Aldrich), CD81 (ab59477, Abcam), BSEP (PA5-27742, Thermo Fisher Scientific), OATP2/8 (ab15441, Abcam), MRP2 (ab3373, Abcam) and SRB1 (ab52629, Abcam).

Further characterization of iPSC-derived hepatocytes was achieved using functional assays. Stored lipids were detected by Oil Red O staining. The hepatocytes were fixed with 4% PFA and incubated in 0.5% Oil red O solution (O1391, Sigma-Aldrich). The cells were rinsed and nuclei were counter-stained with haematoxylin.

Stored glycogen was detected by Periodic Acid Schiff (PAS) staining. The cells were fume-fixed with 4% PFA and permeabilized with 0.1% Triton X-100, following which they were incubated with Periodic acid, and then Schiff’s solution. The cells were counter-stained with haematoxylin. Low-density lipoprotein uptake and regulation was assessed by LDL Uptake Assay Kit (ab133127, Abcam) according to the manufacturer’s protocol. Albumin and Alpha-1 Anti trypsin secretion was measured by human albumin ELISAKit (ab108788, Abcam) and Alpha 1 Antitrypsin ELISA Kit (ab108799 Abcam), respectively, using culture supernatants of the cells, according to the manufacturer’s protocol. Bile transporter activity was assessed by indocyanine green (ICG) (I2633, Sigma-Aldrich) uptake and release through the Liver-Specific organic anion Transporter-1 (LST1). Cellular uptake and release of ICG was examined in 1 h and overnight incubation, respectively. Cytochrome P450 (CYP) activity was measured by P450-Glo CYP3A4 Assay (V9001, Promega, Madison WI, USA) with non-lytic cell-based assay according to the manufacture’s protocol. The hepatocytes were transferred into 96-well plate at 30,000 cells/well for infection assays.

### Infection of the hepatocytes (PHH, HC04, iPSCs derived from vivax patient and non-patient) with the *P. vivax* sporozoites

*Plasmodium vivax* sporozoites were prepared by dissecting the infective salivary gland of *Anopheles stephensi* on day 14 post membrane feeding. The *P. vivax* sporozoites were diluted in dissection medium (without serum) and counted using haemocytometer. The ratio of either 2 sporozoite for 1 hepatocyte (2:1) or 1 sporozoite for 1 hepatocyte (1:1) was typically used for infection assays, unless mentioned otherwise. Accordingly, the desired number of sporozoites were adjusted in appropriate volume of dissection medium and added per well in either 384 well or 96 well plate formats having the respective hepatocyte cells (PHH, HC04 or iPSCs derived hepatocytes). Additionally, different hepatoma cell lines, namely HepG2, Chang liver, WRL-68 and PLC/PRF were also cultured and assessed for their susceptibility to *P. vivax* sporozoite infection. All cell types were seeded 24 h prior to infection with sporozoites and checked for cell confluency prior to infection. After infecting the respective wells with *P. vivax* sporozoites, the culture plates were centrifuged at 200 g for 3 min and incubated at 37 °C, 5% CO2 for 4 h followed by washing infected hepatocytes with complete medium to remove uninvaded sporozoites and salivary gland debris. Next, the *P. vivax* sporozoite infected cultures were maintained at 37 °C, 5% CO_2_ with everyday media change until fixed by 4% PFA for 15 min at room temperature at defined time points for respective set of experiments.

### Immunofluorescence assays

The *P. vivax* infection in the fixed hepatocyte cells were analysed by immunostaining to detect the presence of small and large forms of the parasite, using *P. vivax* specific antibodies, namely *Plasmodium* HSP70 and PvUIS4. DAPI was used as nuclear stain. All the images were acquired in Nikon Ti2 series microscope at 40X magnification and the image analysis was performed using FIJI (ImageJ). Hepatocytes were considered infected if the following criteria were met, a) positive signal for UIS4 antibodies showing the characteristic ring-like shape of *P. vivax* liver stage forms, b) presence of the signal inside the hepatocyte cell, as assessed by bright field images, c) DAPI staining for the parasite nuclei, which gives a distinctive staining pattern compared to host nucleus.

### Compound treatment

Compounds were added once per day along with the media (MEM/F12 +10% FBS + 1X Anti Anti + Amikacin 200 μg/ml) until four days after infection starting from 4 h after infection. MMV390048 [32] was used in the concentration of 10 µM whereas Torin was used in the concentration of 250 nM [33].

### Statistical analyses

*Plasmodium vivax* infection experiments were performed in three or more independent batches consisting of differentiated hepatocytes from a minimum of three iPSC clones/patient along with ES- and non-patient iPSC-derived hepatocytes and HCO4 hepatoma cell line in triplicate or more for each condition. Data from representative batches are presented. For all comparisons between two conditions, statistical significance was assessed by Student’s *t* test were performed.

## Results

### Production of *Plasmodium vivax* sporozoites

**Fig. 1:**
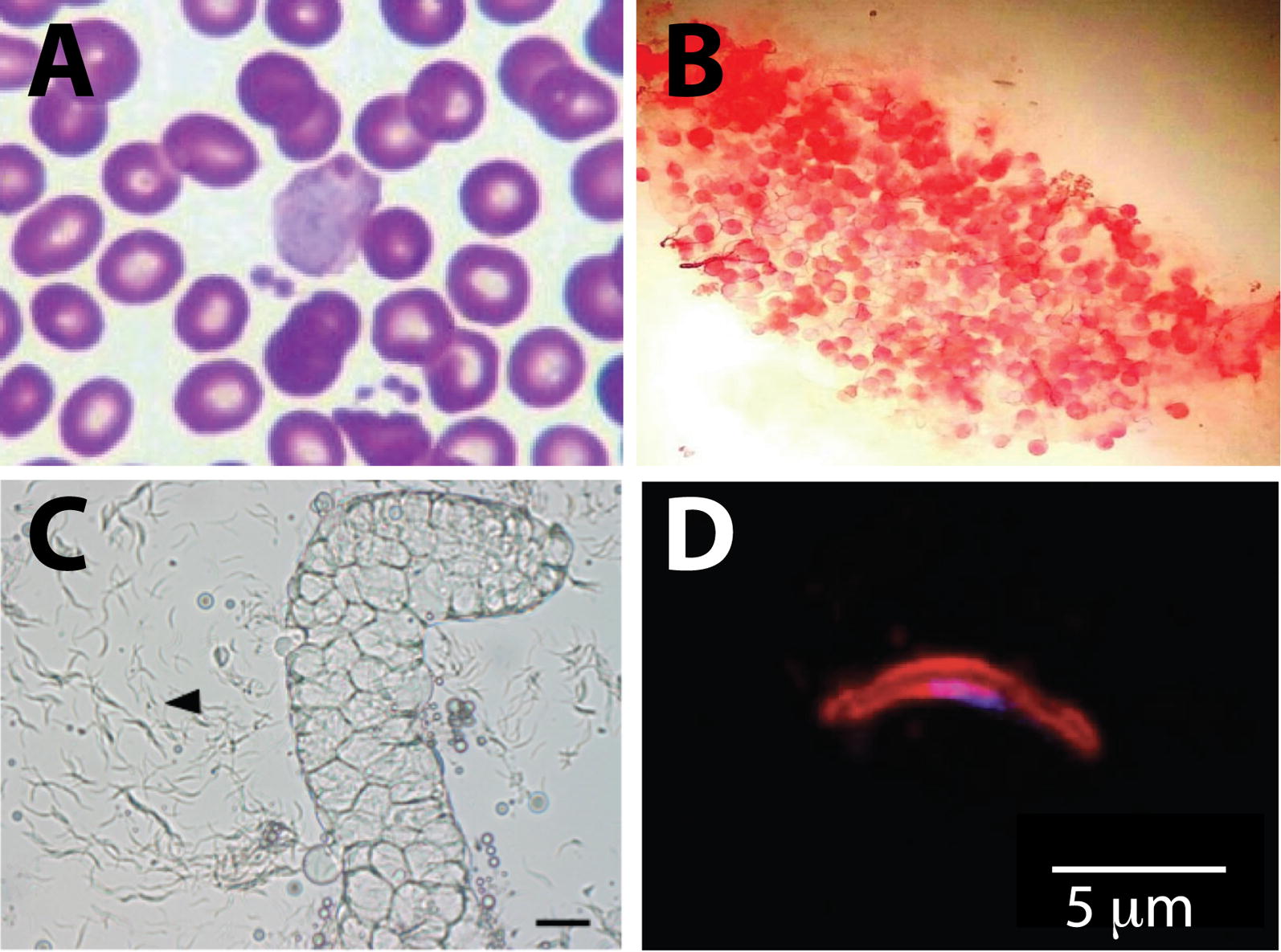
Generation of *P. vivax* salivary gland sporozoites. **a** Thin blood smear from a *P. vivax* infected patient stained with Giemsa, showing the presence of a *P. vivax* gametocyte. **b** Mercurochrome stained midguts from mosquitoes fed with *P. vivax* infected blood, dissected 7 days post infection showing the presence of oocysts. **c** Salivary gland dissected from *P. vivax* infected mosquito 14 days post infection. Image shows release of *P. vivax* sporozoites, as indicated by an arrowhead. Scale bar is 20 μm. **d** Salivary gland sporozoite dissected from *P. vivax* infected mosquito immunostained for pvCSP

*Anopheles stephensi*, one of the six epidemiologically important vectors for malaria transmission out of 58 *Anopheles* species in India, and the key vector for malaria in urban areas including the endemic region of Mangalore [34], was used for infection and production of *P. vivax* sporozoites. Blood from *P. vivax* mono-infected patients was confirmed for the presence of gametocytes (Fig. 1a) and immediately fed to 3-4 days old female *An. stephensi*. The development of *P. vivax* in the mosquitoes was confirmed by detection of oocysts in the mid gut 8–10 days post membrane feeding (Fig. 1b). 14 days post blood feeding, salivary glands of infected mosquitoes were dissected by mechanical rupture to release *P. vivax* sporozoites (Fig. 1c). The identity of sporozoites was confirmed by immuno-staining with *P. vivax* specific anti-CSP antibody (Fig. 1d).

### In vitro infection with *P. vivax* sporozoites

**Fig. 2:**
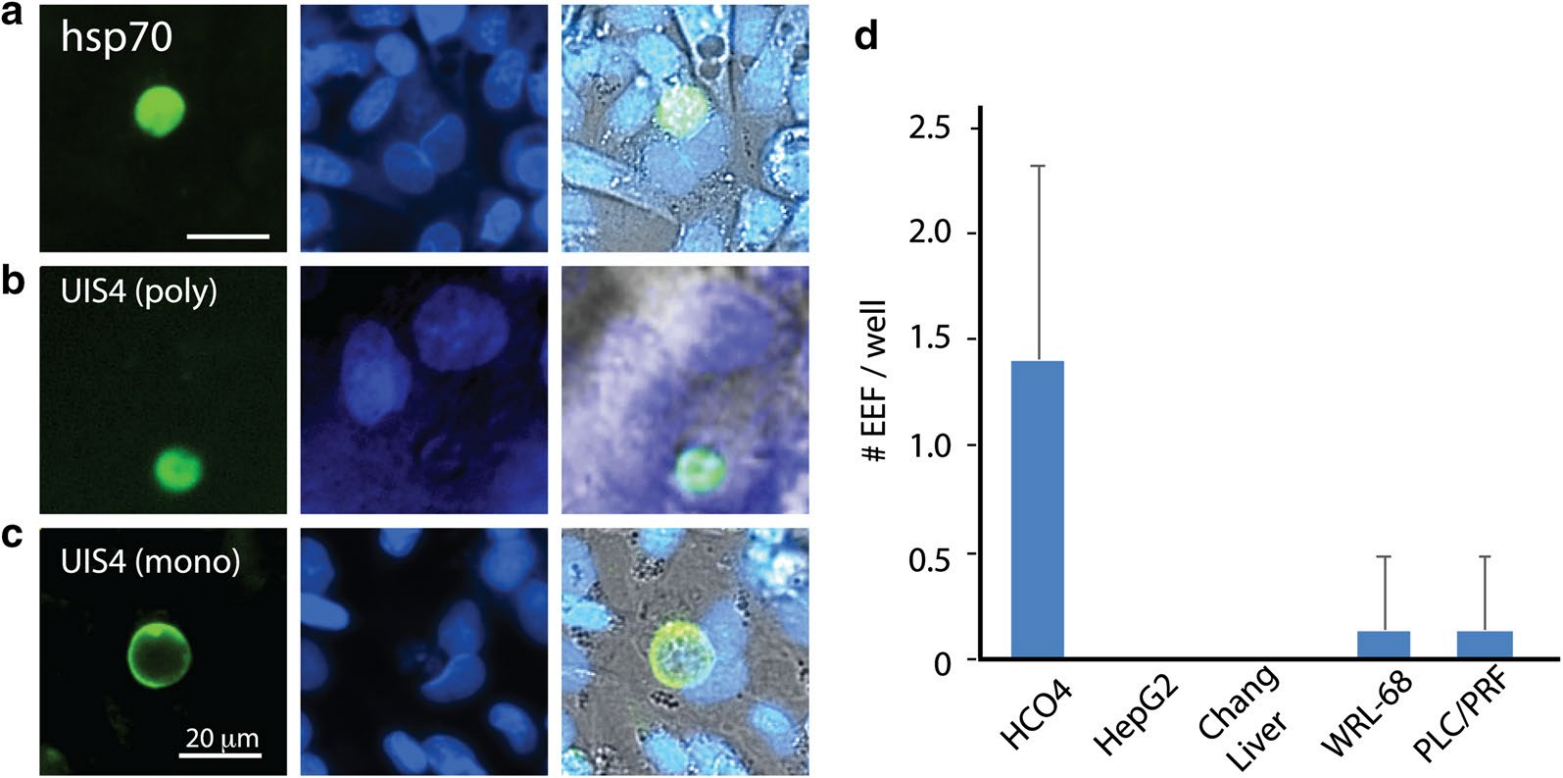
Comparison of hepatoma cells for infectivity with *P. vivax* sporozoites. **a**–**c** HCO4 cells were infected with *P. vivax* sporozoites, fixed at 5 days post infection and immunostained with anti-hsp70 (**a**), anti-UIS4polyclonal (**b**) or anti-UIS4 monoclonal (**c**) antibodies. **d** Quantification of number of EEF’s observed in different cell lines infected (HCO4, HepG2, Chang Liver, WRL-68 and PLC/PRF) with *P. vivax* sporozoites. Data averaged from 6 wells per cell line from a 96 well plate. Error bars are standard deviation between wells. Data representative of 3 independent infections

Next, the infectivity of *P. vivax* sporozoites in hepatoma cells was assessed. HCO4 cells have been successfully used for *P. vivax* infection from Thai [14] and Peruvian isolates [17] and were, therefore, first investigated with the Indian *P. vivax* isolates. Cells were infected with *P. vivax* sporozoites as described in Methods, fixed at 4 days post infection and immunostained with different antibodies recognizing *P. vivax* liver stage forms, alternatively called as exo-erythrocytic forms (EEF). The results show positive staining for hsp70 (Fig. 2a), UIS4 (polyclonal) (Fig. 2b), and UIS4 (monoclonal) (Fig. 2c). For subsequent assays, either a combination of UIS4 (polyclonal) and hsp70, or pv UIS4 (monoclonal), was used, and number of EEF’s obtained per well of 384 well plate counted. Across multiple experiments and replicates, low number of infected cells was observed consistently (Fig. 2d). Next, different conditions were explored to optimize the assay and assess if infectivity rates can be improved. Towards this, HCO4 cells were infected with different multiplicities of infection (MOI) of infection (Additional file 1: Fig. S1A), increasing the duration of infection (Additional file 1: Fig. S1A) and washing patient blood with serum prior to membrane feeding (Additional file 1: Fig. S1B). However, the different conditions tested did not result in increase in infectivity. Next, a panel of different hepatoma-like cells (HepG2, Chang Liver, WRL-68, PLC/PFR) were tested and EEF numbers assessed at either 4 or 5 days post infection to examine if they showed improved infectivity. The results (Fig. 2d) show that the different cell lines tested did not show improvement in infectivity.

**Fig. 3:**
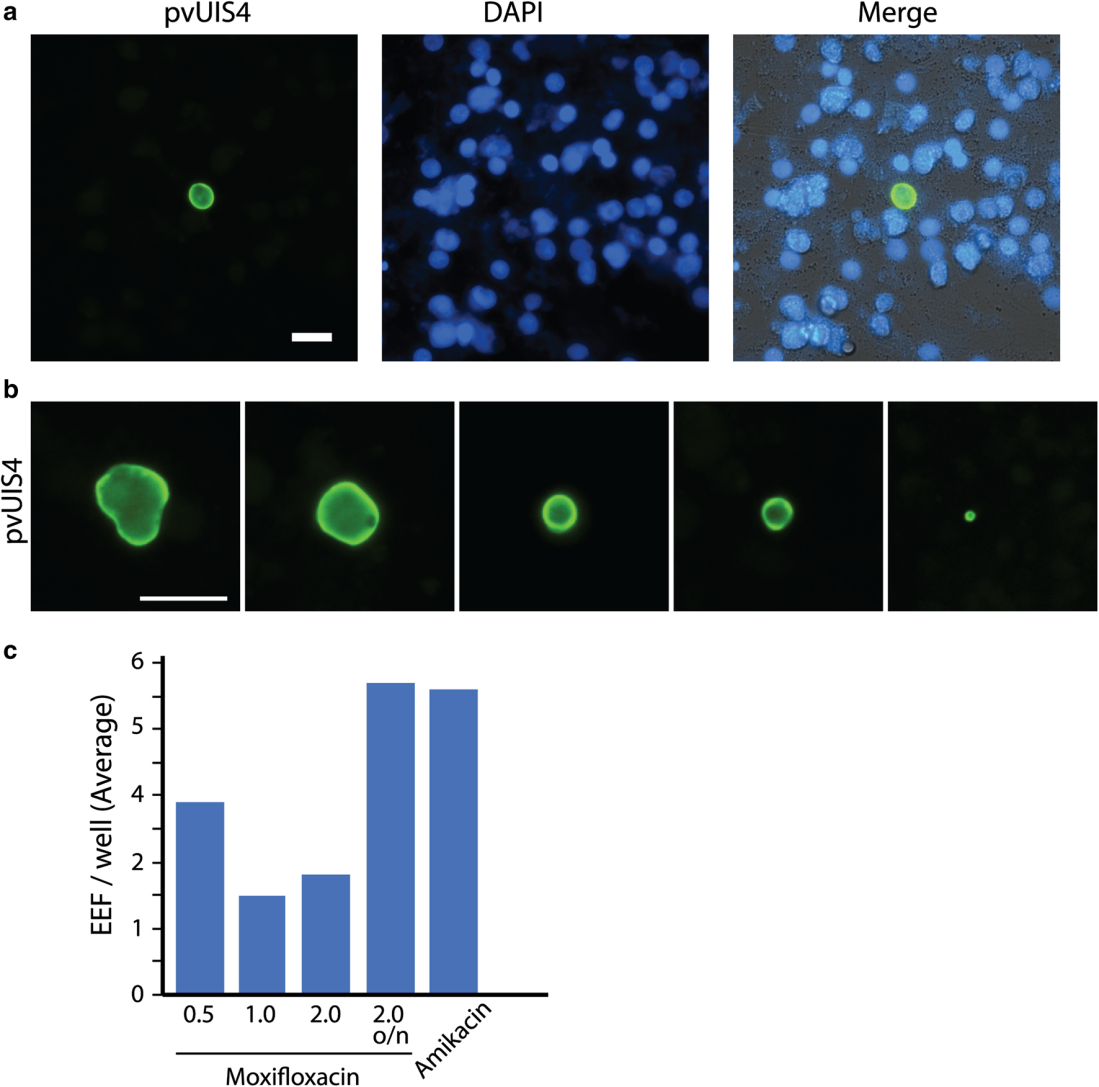
Infectivity of primary human hepatocytes (PHH) with *P. vivax* sporozoites. **a** Representative image from primary human hepatocytes infected with *P. vivax* sporozoites, fixed at 7 days post infection and immuno stained for pvUIS4 antibody. **b** Panels showing pvEEF’s of different sizes from the same infection. For A, B, scale bar is 25 μm. **c** Quantification of number of EEF per well in primary human hepatocytes infected with *P. vivax* sporozoites under different culture conditions: Addition of amikacin or various concentrations of moxifloxacin to basic Pen-Strep-Neo containing media during sporozoite infection 4 h or o/n (overnight) enabled long term sterile hepatocyte cultures with demonstrable EEFs (Amikacin200 μg/ml, n = 20wells; Moxifloxacin 0.5, 1.0 and 2 μg/ml, n = 30 wells each and Moxifloxacin o/n treatment, n = 60 wells)

Primary human hepatocytes (PHH) has been successfully used as a model for *P. vivax* liver stage infections from South East Asian vivax isolates [18–21]. Next, PHH was tested for its ability to support infection from Indian *P. vivax* isolates following previously established protocols [18], and infection rate assessed at 4–7 days post infection following staining with UIS4 monoclonal antibody. The results (Fig. 3a) shows that primary human hepatocytes are infected with Indian *P. vivax* sporozoites. Indeed, EEF’s of different sizes can be observed in primary human hepatocytes (Fig. 3b). However, the number of EEF’s per well remained low and did not show appreciable changes under different antibiotic treatment conditions tested (Fig. 3c).

### Generation of hepatocytes from vivax malaria patient blood monocytes

In an effort to improve the infectivity, the option of using iPSC derived hepatocytes, which have also been shown to be a permissive platform for *P. vivax* liver stage infection [25], was explored. Patients from vivax endemic region report a wide spectrum of severity of clinical manifestations [2], suggesting that some individuals might be more susceptible for vivax malaria, possibly depending on genetic factors. Hepatocytes from these patients could be more permissive for vivax infection in vitro. Hence, in addition to the standard embryonic stem cell (ESC) derived hepatocytes, hepatocytes were generated from selected vivax patients from blood monocytes using stem cell technology.

**Fig. 4:**
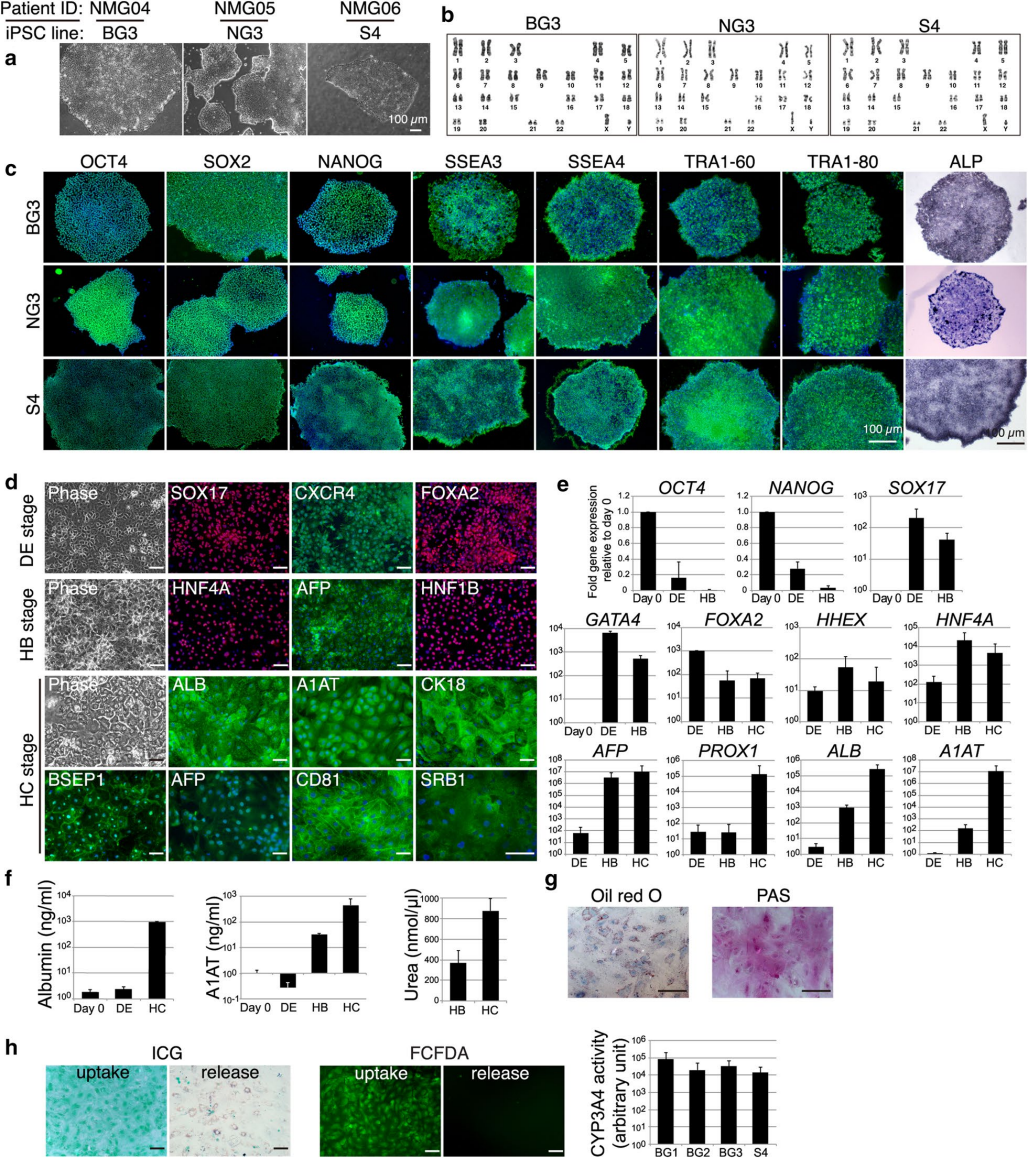
Generation and characterization of monocyte derived hepatocytes from *P. vivax* infected patients. **a** Representative image of emerging iPSC colony obtained from three different *P. vivax* patient PBMCs at day 12 of the protocol. **b** Karyotyping result of the indicated iPSC lines derived from three different vivax patient. **c** Representative images of indicated pluripotency marker expression in iPSC’s derived from monocytes of three different vivax patients assessed by antibody staining and ALP activity staining. DAPI (blue) was used as a nuclear stain in fluorescence images. Scale bar is 100 µm. **d** Representative images of phase contrast and respective marker expression based on antibody staining from DE, HB and HE stages from one of the patient iPSC derived hepatocyte. Scale bar is 100 μm. Representative images of phase contrast and immunostaining image of HB stage from one of the patient iPSC derived hepatocyte. Green signals indicate the relevant markers, and blue signals indicate DAPI nuclear staining. Scale bar is 100 µm. **e** RT-qPCR gene expression analysis of the indicated pluripotency and differentiation markers (n = 3). Error bars indicate standard deviation. **f** Concentration of albumin, alpha 1 antitrypsin (A1AT) and urea produced in the culture medium of each differentiation stage of patient derived iPSC to hepatocytes. Error bars indicate standard deviation (n = 3, 4 and 4, respectively). **g** Representative image of Oil red O and PAS staining of the differentiated hepatocyte from a patient derived iPSC. **h** Bright field image of ICG uptake post washing after 30 min treatment and release after 60 min post wash from a patient iPSC derived hepatocyte. CYP3A4 activity of the hepatocytes differentiated from the different patient derived iPSC lines

Malaria patients from endemic areas in Mangalore were screened for *P. vivax* mono-infection using the criteria described in the Methods section. Following patient screening and selection, peripheral blood mononuclear cells (PBMCs) from three patients were obtained using density gradient centrifugation, enriched for CD34+ haematopoietic progenitor cells and transfected with reprogramming factors to generate iPSC. At least three morphologically relevant, pluripotent marker-positive, karyotypically normal and stable iPSC lines/patient were established from three *P. vivax* patients PBMCs for further hepatic differentiation (Fig. 4a–c). Similarly, a non-patient iPSC was derived and characterized, as described previously [31]. Hepatocytes were differentiated from the iPSC lines using a 30-day protocol based on previously reported literature [35], but with several modifications. During this differentiation process, iPSCs gradually lost pluripotency marker *OCT4* and *NANOG* expression (Fig. 4e) and adopted a definitive endoderm (DE) fate by day 5, wherein > 95% of cells started expressing mesoendodermal marker such as CXCR4 and *GATA4* and DE markers such as FOXA2 and SOX17(Fig. 4d, e). By day 10, the cells started to demonstrate morphologies similar to hepatic-specified endoderm and hepatoblasts expressing early hepatic markers such as HNF4A, AFP, HNF1B and HHEX (Fig. 4 d, e). Immature hepatocyte-like cells became visible by day 20 and further transition into mature hepatocyte-like cells exhibiting similarities with primary hepatocytes, including bi-nucleate cells with prominent nucleoli, by day 30 (Fig. 4d). At this stage, the cells express hepatocyte marker genes such as *PROX1*, *ALB* and *A1AT* (Fig. 4e) and > 70% cells stained positive for mature hepatocyte markers tested, namely, ALB, A1AT, BSEP1 and CK18 (Fig. 4d). As expected, the cells were capable of synthesizing and secreting Albumin and A1AT as well as producing urea (Fig. 4f). Oil red O and PAS staining revealed > 80% cells displaying storage of neutral triglycerides and lipids, and storage of polysaccharide glycogens and glycoproteins (Fig. 4g). Moreover, the cells could actively transport and uptake the dichlorofluorescein diacetate (DCFDA) dye and metabolize indocyanine green (ICG) and cytochrome P450 3A4 (CYP3A4) substrates, indicating the presence of functional transporters and metabolic activity (Fig. 4h). Most importantly, the mature hepatocytes were positive for the known *P. vivax* entry molecule SRB1 [36] as well as CD81 (Fig. 4d). Thus, hepatocytes obtained from *P. vivax* patient iPSCs are functional as they gather all the normal machinery and membranes receptors of human primary hepatocytes.

### *Plasmodium vivax* infection of patient iPSC derived hepatocytes

**Fig. 5:**
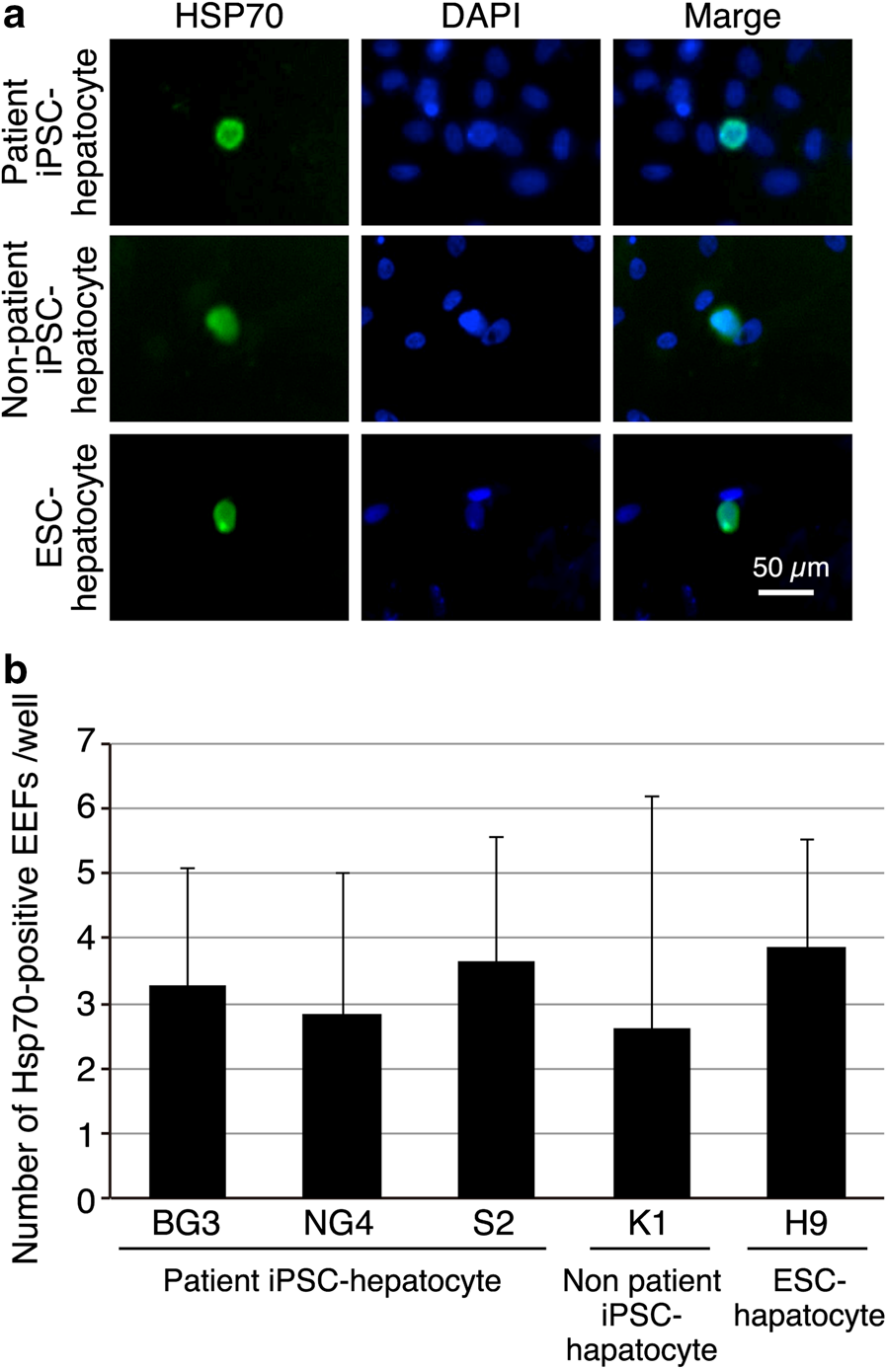
Infectivity of iPSC derived hepatocytes from *P. vivax* infected patients. **a** Detection of *P. vivax* EEFs in patient iPSC-derived hepatocytes with *P. vivax* sporozoites at 4 days post infection. Green signal indicates immuno-staining with *P. vivax*-specific anti-HSP70 antibody, blue colour indicates DAPI nuclear staining. Scale bar is 50 μm. **b** Quantitation of number of EEF’s per well for different patient iPSC derived hepatocytes. K1 and H9 are hepatocytes derived from healthy volunteers and ESC, respectively. Error bar represents standard deviation of replicates. Data are pooled from 6 wells per condition, and are representative of three independent infections

The hepatocytes derived from iPSC’s from three vivax patients with *P. vivax* sporozoites, along with hepatocyte derived from non-patient iPSC as well as human embryonic stem cell (H9), were infected with *P. vivax* sporozoites. Cells were fixed at day 5 post-infection and infection assessed by immunostaining. The results show that *P. vivax* EEF’s can be detected in all the iPSC derived hepatocytes (Fig. 5a), however there was no significant differences in the infectivity rate between hepatocytes derived from *P. vivax* patient iPSCs, non-patient iPSCs and ESCs. Moreover, the infectivity was also not different from the other systems tested previously. Collectively, these results from multiple platforms demonstrate the feasibility of detecting vivax liver stage forms from Indian *P. vivax* isolates, but at a rate that is currently not compatible for drug screening campaign.

### Characterization of *P. vivax* liver stage forms

**Fig. 6:**
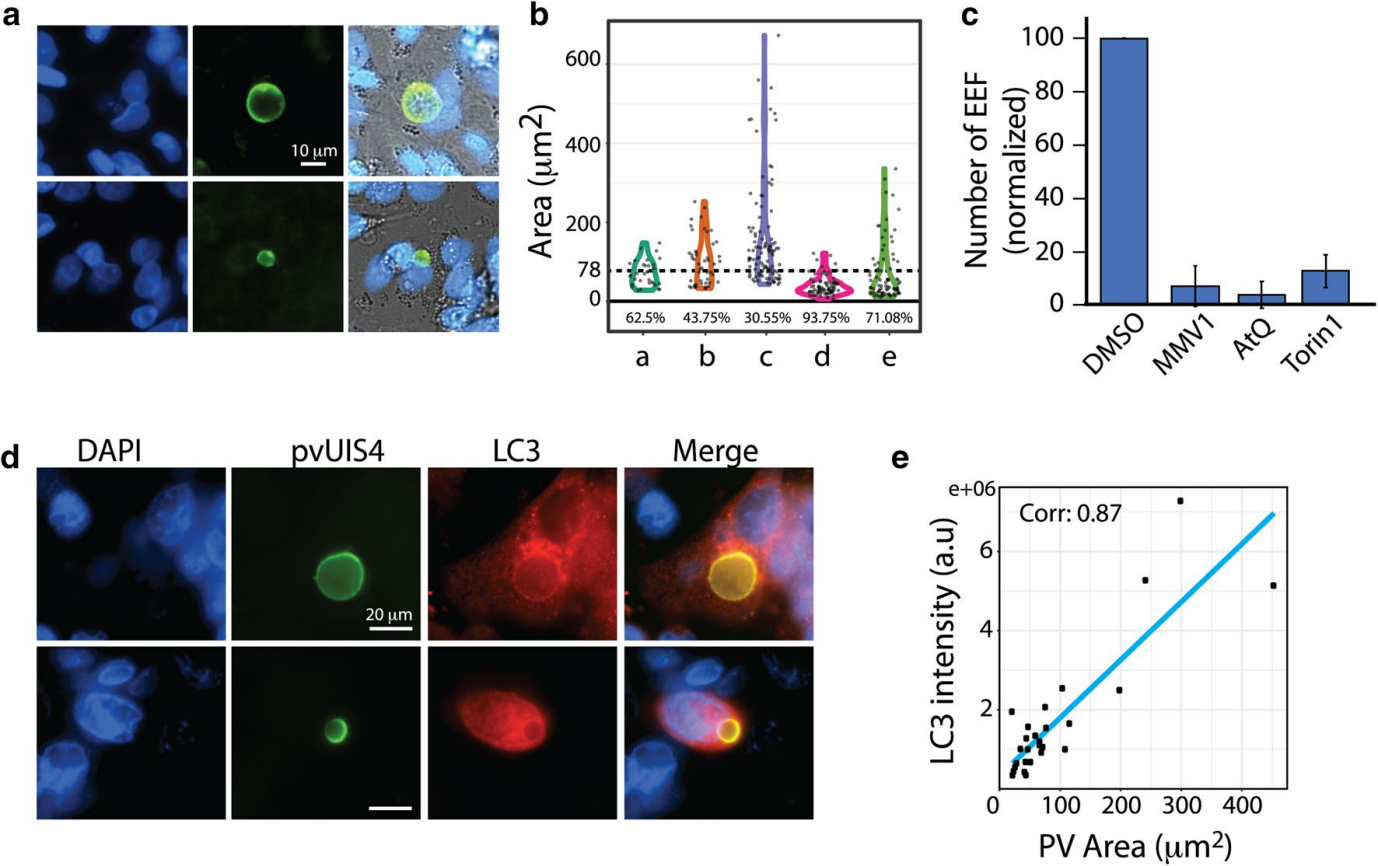
Characterization of liver stage EEF from Indian *P. vivax* strains. **a** Representative images of *P. vivax* infected HCO4 cells stained with anti UIS4 antibody, 5 days post infection. EEF’s of different sizes are seen. **b** Quantification of EEF area from HCO4 cells infected with *P. vivax* sporozoites and fixed days 5 post infection. Datapoints represent individual EEF, results pooled from multiple wells of the same infection. **a**–**e**, represent the area distribution from three independent infections. **a**–**d** is from HCO4 cells, **e** is from primary human hepatocytes. Dashed line is at 78 μm^2^, fraction of parasites lesser than 78 μm^2^ per infection is indicated in the boxed area. **c** Reduction in the EEF numbers upon drug treatment. *P. vivax* sporozoite infected HCO4 cells are treated with indicated compounds daily for 4 days post infection, fixed, immunostained for pvUIS4 and number of EEF’s assessed. Data pooled from 30 wells of 384 well plate per condition. ‘MMV1’ denotes MMV390048, a PI4K inhibitor with known activity against liver stage *P. vivax* forms. Results averaged from three independent infections. Data normalized to DMSO of each infection, error bars indicate standard deviations. **d***P. vivax* PVM is positive for LC3. *P. vivax* infected HCO4 cells were immunostained for UIS4 and the host autophagy marker LC3. EEF’s corresponding to both small and large forms from the same infection are shown. Results representative of at least three independent infections. Scale bar is 20 μm. **e** Scatter plot showing the intensity of LC3 per EEF spanning different size ranges of EEF shows a linear relationship. Corr corresponds to correlation value

Since low infectivity was observed in all model systems tested, data were pooled from multiple wells to perform the first characterization of vivax liver stage forms from Indian isolates. First, the parasite area in different infections was quantified. Parasites of different sizes in the same infection was frequently observed, suggesting significant heterogeneity in the parasite development (Fig. 6a, b). Distribution of the parasite areas across different infections (Fig. 6b) shows a broad distribution and variance across different infections, with a significant proportion of events lesser than 78 μm^2^, that corresponds to diameter of 10 μm, typically considered as small forms of *P. vivax* liver stage [15, 20]. In several instances, large multinucleated forms corresponding to schizonts were observed, (Fig. 6a, d, top panels).

### Effect of compound treatments on *P. vivax* infection

The low level of infectivity precluded the possibility of using these platforms for high throughput drug screening. However, the utility of the platform to validate selected compounds against Indian liver stage vivax forms was tested using known compounds such as Atavaquone and MMV390048, a PI4K inhibitor with anti-malarial activity [32] in a prophylactic mode by adding the compounds immediately after infection with daily changes for 5 days. Torin1 was used as additional control, as it has been shown to be effective in clearing the liver stage forms of *Plasmodium berghei* [33] in prophylactic mode. All compounds were used in a single concentration. Data from multiple infected cells from a group of wells were pooled to determine the effect of compounds on parasite development in HCO4 cells. The results (Fig. 6c) shows that, as expected, atovaquone significantly inhibited the development of liver stage forms. Similarly, Torin1 as well as MMV390048 showed strong inhibition (Fig. 6c). These results show that the platforms established here, while not suitable for high throughput screening, might be useful to test promising candidates against parasites of Indian origin. However, further testing including dose–response analysis will be necessary to substantiate these tests.

Finally, the potential of the platforms established to address the biology of vivax liver stage forms was tested. Previous reports of involvement of host autophagy machinery during liver stage *P. vivax* infection [37, 38] prompted us to assess the localization of the host autophagy molecule LC3 on *P. vivax* parasitophorous vacuole membrane (PVM). Towards this, HC04 cells infected with *P. vivax* sporozoites and fixed at 5 days post infection was immunostained for UIS4 and LC3. Confocal laser scanning microscopy revealed that LC3 strongly co-localized with UIS4, in both small and large forms (Fig. 6c). To confirm this, total intensity of LC3 on the PVM of individual liver stage forms was plotted. The results (Fig. 6d) shows a linear relationship between the parasite area and LC3 intensity.

## Discussion

In this study, multiple platforms to assay *P. vivax* liver-stage forms from Indian vivax isolates have been established. These include different hepatoma cell lines, primary human hepatocytes as well as hepatocytes differentiated from iPSC’s derived from Indian *P. vivax* patients. These studies demonstrate a proof-of-concept for detection and characterization of *P. vivax* liver stage forms and provide a platform for establishing a robust liver stage assay in a *P. vivax* endemic region which could later on be used for high throughput screening of chemical libraries.

The burden of malaria in India is very complex, owing to its highly variable eco-epidemiological profile, flexible transmission factors, multiple *Plasmodium* species and *Anopheles* vectors [39]. Specifically, *P. vivax* epidemiology across India varies considerably due to multiple relapse phenotypes with varying latency periods, and a dynamic disease profile due to reestablishment of the disease in eliminated areas owing to hypnozoite reservoirs with transmission potential following eradication. Most information in literature on the *P. vivax* liver stage characteristics such as the rates and proportion of hypnozoites, relapse patterns, transcriptomics, susceptibility to small molecules are from *P. vivax* isolates from endemic areas of south-east Asia and South America [12, 17, 18, 20, 21, 25, 40, 41]. India accounts for ~ 50% of vivax cases world-wide [3], however while recent studies have shed light on *P. vivax* oocyst and sporozoite stages in mosquitoes [42, 43] no information is available about the characteristics of liver stage forms from Indian vivax isolates. This report is the first documentation of the liver stage characteristics from Indian isolates of *P. vivax.*

In this study, different host cell systems such as the hepatoma-like cell lines (HCO4, HepG2, Chang liver, WRL-68 and PLC/PRF), Primary human hepatocytes (PHH) as well as hepatocytes differentiated from iPSC generated from vivax infected patient PBMC have been used. The systems used are different from each other, hence direct comparison between them is not possible. However, these results show that across the multiple systems employed, low infection rates were consistently observed. The low infection rates in all the systems tested precludes drug screening, however, the platform and protocols established could allow testing of individual candidate’s efficacy from other screening campaigns against Indian *P. vivax* isolates, as well as study of parasite biology.

iPSC derived hepatocytes were explored as a host system in an effort to improve infectivity. Hepatocytes were differentiated from iPSC’s derived from vivax infected patients, with a reasoning that combination of *P. vivax* parasite and a person with confirmed *P. vivax* malaria mono-infection in same endemic area could harbor an increased permissiveness towards infection of the hepatocytes. Therefore, a liver stage assay utilizing components from same spatiotemporal location, i.e. hepatocytes from *P. vivax* mono-infected patient-derived iPSCs, *P. vivax* isolates from vivax patients and Anopheles mosquitoes was developed. Although the *P. vivax* sporozoite infection of *P. vivax* patient iPSC-derived hepatocytes could be detected at day 5 post-infection, the infectivity rates were not significantly different between patient-derived, non-patient-derived and ESC-derived hepatocytes. Similarly testing of *P. vivax* sporozoites on other assay platforms such as HCO4 cells and PHH yielded low infectivity. There are several possibilities to explain the low infectivity observed in this study. First, although the source of parasitaemia detected in an individual in an endemic setting can be attributed to a specific *Plasmodium* species, the possibility of a mixed infection, or influence from recent past infections, cannot be entirely ruled out. Second, the sporozoites could be inherently more fragile compared to other geographical strains, which could reflect in reduced viability post dissection. Third, the unidentified immunological status of the *P. vivax* patients from endemic area might alter the infection rates detected in the study. Intrinsic differences in the Indian vivax strains compared to other geographical regions cannot be ruled out. Indian vivax strains show differences in phenotypic traits such as relapse patterns, which vary both geographically and temporally within the country, as well as clinical profiles and drug response [44]. Further, vivax malaria infections in India are characterized by poly-clonality [45, 46] with individual markers showing distinctive patterns compared to other geographical locations [44]. The contribution of these factors, particularly genetic diversity and temporal phenotypic profiles, towards the liver stage infectivity needs to be systematically assessed. Further, direct comparison between Indian and South-East Asian strains under the same experimental setting for liver stage assay could help identify potential experimental or other systematic differences. Finally, adapting novel emerging platforms [18–20, 40] to liver stage assays with Indian vivax strains could help in improving infectivity rates.

## Conclusion

In this study, different platforms to detect liver stage *P. vivax* forms from Indian *P. vivax* clinical isolates have been developed. While both large and small size forms were observed, the low number of liver stage forms observed in all the conditions tested precludes drug screening. Further optimization of assay conditions will be needed to improve infectivity. However, the assay could be readily used for study of vivax liver stage biology and compare with isolates from other regions.

## Supplementary information

**Additional file 1: Fig. S1.** Optimization of *P. vivax* sporozoite infection in HCO4 cells. A Testing different multiplicity of infection (MOI), duration of infection. HCO4 cells were infected with the indicated number of sporozoites per well for either 1 or 4 h. Infection was assessed by immunostaining for pvUIS4. Number of EEF’s per well were counted by microscopy. Results are representative of three independent infections. Error bars represent standard deviations from 30 wells per condition from a 384 well plate. B Blood from vivax patients were either washed in AB + serum, or not, before feeding to mosquitoes. Sporozoites obtained from these mosquitoes were used for infection in HCO4 cells, and number of EEF’s per well counted as described before. Results are representative of two independent infections. Error bars represent standard deviations from 30 wells per condition from a 384 well plate.

## Data Availability

The datasets during and/or analysed during the current study available from the corresponding author(s) on reasonable request
