# Supplementary figures and images for: Plasmodium vivax liver stage assay platforms using Indian clinical isolates
Source: Malar J. 2020 Jun 22;19:214. doi: 10.1186/s12936-020-03284-8 (PMC7310233; doi:10.1186/s12936-020-03284-8)

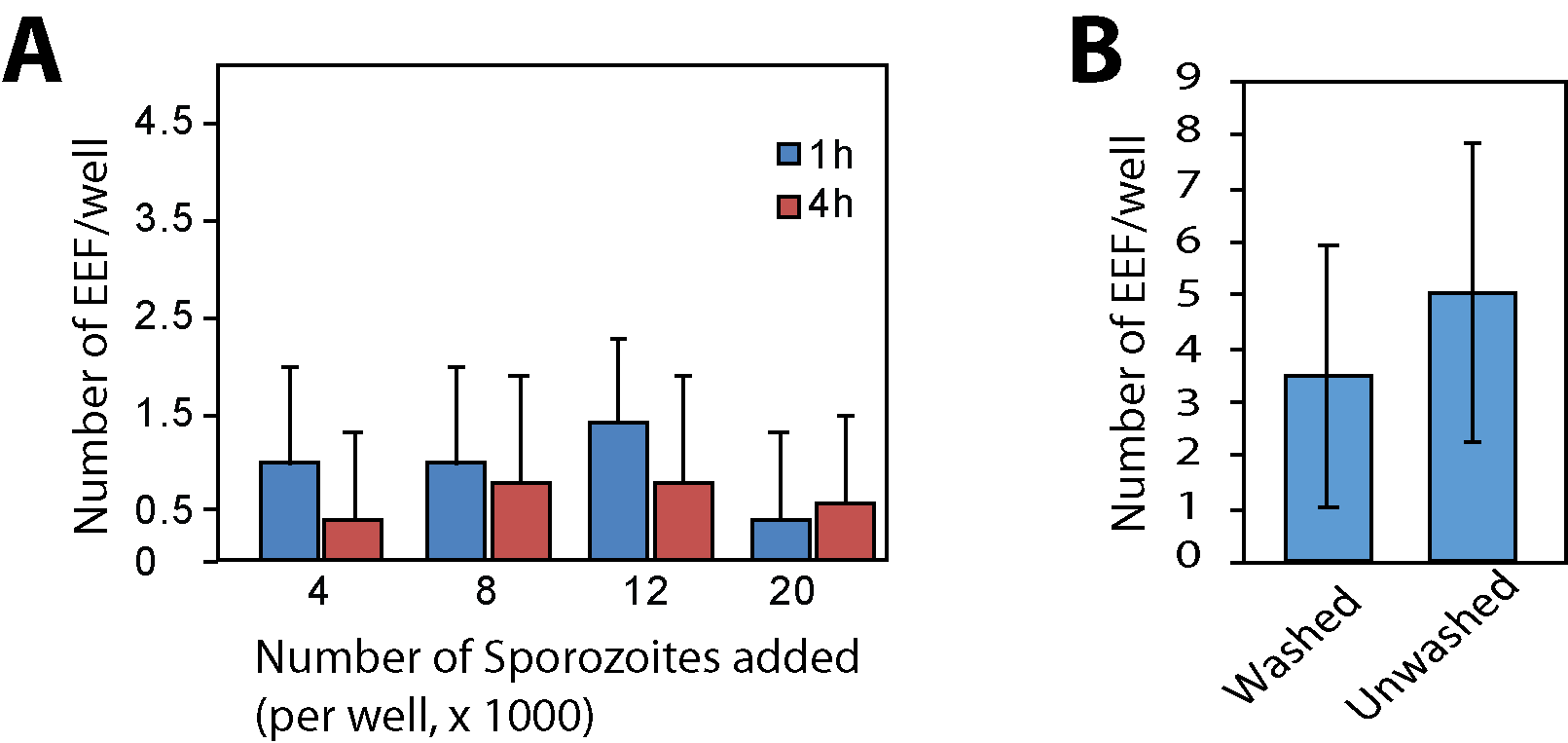

Supplement: Supplementary file 1 — Additional file 1: Fig. S1. Optimization of P. vivax sporozoite infection in HCO4 cells. A Testing different multiplicity of infection (MOI), duration of infection. HCO4 cells were infected with the indicated number of sporozoites per well for either 1 or 4 h. Infection was assessed by immunostaining for pvUIS4. Number of EEF’s per well were counted by microscopy. Results are representative of three independent infections. Error bars represent standard deviations from 30 wells per condition from a 384 well plate. B Blood from vivax patients were either washed in AB + serum, or not, before feeding to mosquitoes. Sporozoites obtained from these mosquitoes were used for infection in HCO4 cells, and number of EEF’s per well counted as described before. Results are representative of two independent infections. Error bars represent standard deviations from 30 wells per condition from a 384 well plate. [file 12936_2020_3284_MOESM1_ESM.tif]
